# Effects of Tannic Acid, Green Tea and Red Wine on hERG Channels Expressed in HEK293 Cells

**DOI:** 10.1371/journal.pone.0143797

**Published:** 2015-12-01

**Authors:** Xi Chu, Yusong Guo, Bingyuan Xu, Wenya Li, Yue Lin, Xiaorun Sun, Chunhua Ding, Xuan Zhang

**Affiliations:** 1 Department of Pharmacology, Hebei University of Chinese Medicine, Shijiazhuang, China; 2 Arrhythmia Center, Guangdong Provincial Hospital of Chinese Medicine, Guangzhou, China; 3 Department of Pharmacy, The Fourth Hospital of Hebei Medical University, Shijiazhuang, China; 4 Department of Orthopaedic Trauma Dept. 2, The Third Hospital of Shijiazhuang, Shijiazhuang, China; 5 Department of Biochemistry, Hebei University of Chinese Medicine, Shijiazhuang, China; 6 Department of Physiology, Hebei University of Chinese Medicine, Shijiazhuang, China; Rutgers, the State University of New Jersey, UNITED STATES

## Abstract

Tannic acid presents in varying concentrations in plant foods, and in relatively high concentrations in green teas and red wines. Human *ether-à-go-go*-related gene (hERG) channels expressed in multiple tissues (e.g. heart, neurons, smooth muscle and cancer cells), and play important roles in modulating cardiac action potential repolarization and tumor cell biology. The present study investigated the effects of tannic acid, green teas and red wines on hERG currents. The effects of tannic acid, teas and red wines on hERG currents stably transfected in HEK293 cells were studied with a perforated patch clamp technique. In this study, we demonstrated that tannic acid inhibited hERG currents with an IC_50_ of 3.4 μM and ~100% inhibition at higher concentrations, and significantly shifted the voltage dependent activation to more positive potentials (Δ23.2 mV). Remarkably, a 100-fold dilution of multiple types of tea (green tea, oolong tea and black tea) or red wine inhibited hERG currents by ~90%, and significantly shifted the voltage dependent activation to more positive potentials (Δ30.8 mV and Δ26.0 mV, respectively). Green tea Lung Ching and red wine inhibited hERG currents, with IC_50_ of 0.04% and 0.19%, respectively. The effects of tannic acid, teas and red wine on hERG currents were irreversible. These results suggest tannic acid is a novel hERG channel blocker and consequently provide a new mechanistic evidence for understanding the effects of tannic acid. They also revealed the potential pharmacological basis of tea- and red wine-induced biology activities.

## Introduction

The human ether-à-go-go-related gene (hERG) conduct the rapid delayed rectifier K^+^ current (I_*Kr*_) and mediate action potential repolarization in the heart, its blockade by drugs can lead to long QT syndrome [1,2]. A growing list of agents with hERG blockade effects have been withdrawn from the market or restricted in their clinical use [3]. On the other hand, hERG channels are not only identificated in the heart, but also in several other tissues (e.g. neurons, smooth muscle and cancer cells), and up-regulation of hERG channel expression has been demonstrated in specific tumors [4]. Recent evidence highlights the role of hERG channels in tumor cell biology (proliferation, invasiveness and neoangiogensis), which implies that hERG may be used as a novel diagnostic and prognostic marker in cancer as well as a target for anti-neoplastic therapies [4].

Tea is one of the most widely consumed beverages in the world. It can be categorized into three types, depending on the level of fermentation, i.e., green (non-fermented), oolong (semi-fermented) and black (full-fermented) tea. Teas and red wines are purported to have a number of beneficial health effects including antithrombotic, anti-inflammatory, and anticancer activities [5,6,7] Epidemiological studies have shown a negative association between tea consumption and the incidence of cancers.

Tannic acid presents in varying concentrations in plant foods, and in relatively high concentrations in red wines and teas [8,9,10,11,12]. For example, the tea plant contains abundant tannic acid (~40 mg/g)[13]. Recently, tannic acid has been associated with anticancer functions in multiple tumor types both *in vitro* and *in vivo* [14,15,16]. Tannic acid and related green tea polyphenols inhibited the mouse mammary tumor virus promoter [17,18]. In vitro, tannic acid inhibited the proliferation of various cancer cell lines and induced cancer cell apoptosis [19,20].

This study provides the first description of tannic acid, teas and red wines on hERG currents which provide a potential molecular mechanism for their known biology activities.

## Materials and Methods

### Cell lines and culture

The HEK293 wild-type cell line was purchased from American Type Culture Collection (ATCC, Maryland, USA). The HEK293 cells stably transfected with hERG were constructed by Yuhong Wang (Department of Pharmacology, Hebei Medical University, China). HEK293 cells stably transfected with hERG were cultured in DMEM supplemented with 10% fetal bovine serum, 1% nonessential amino acids, 600 μg/ml G418 and 1% penicillin/streptomycin in a humidified incubator at 37°C (5% CO_2_). Cells were seeded on glass coverslips in a 24-multiwell plat. Assays were done at 48 h after plating.

### Electrophysiology

Perforated whole-cell patch recordings were performed on HEK293 cells. Recordings were made at room temperature (23~25°C). Pipettes were pulled from borosilicate glass capillaries and had resistances of 1.5~2.5 MΩ when filled with internal solution. Currents were recorded using an Axon patch 200B amplifier and pClamp 10.0 software (Axon Instruments, Foster City, CA, USA), and were filtered at 2 KHz. For perforated patch recording, a pipette was first front-filled with the standard internal solution, then backfilled with the same internal solution containing amphotericin B (250 μg/ml). The external solution consisted of (in mM): 160 NaCl, 2.5 KCl, 2 CaCl_2_, 1 MgCl_2_, 10 HEPES, and 8 glucose, pH 7.4. The internal solution consisted of (in mM): 150 KCl, 5 MgCl_2_, 10 HEPES, pH 7.4.

### Teas and red wines

**Fig 1:**
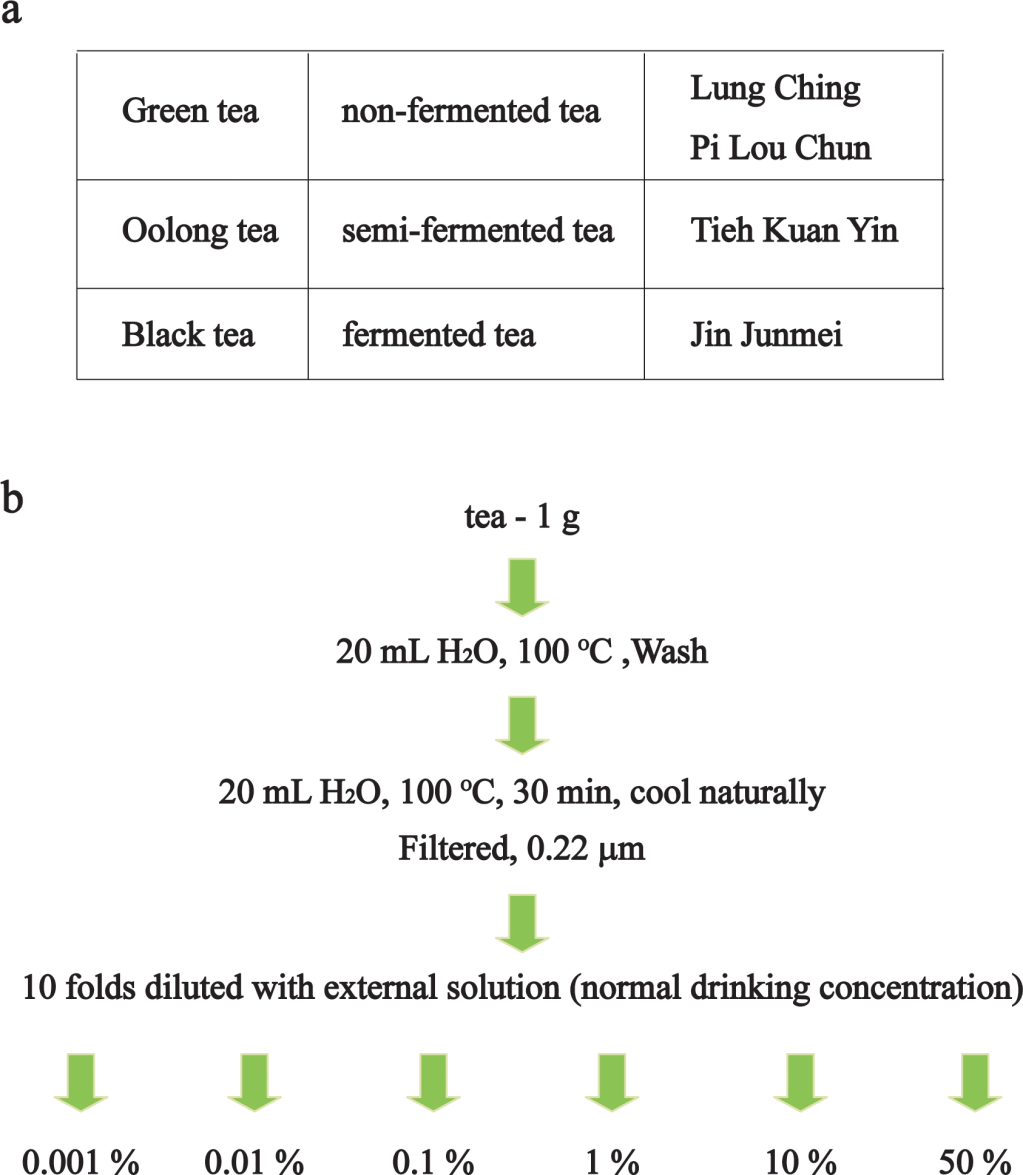
The classification (a) and preparation of teas (b).

Green teas (Lung Ching, Cat No: 1018595518; Pi Lou Chun, Cat No: 1000812899), oolong tea (Tieh Kuan Yin, Cat No: 1000137394), and black tea (Jin Junmei, Cat No: 1002630971) were purchased from Tianfu (Fujian, China). Red wines (Cat No: 996807) were purchased from Great Wall (Hebei, China). Green tea, oolong tea or black tea (1 g) was washed in 20 ml 100°C water, and incubated in 20 ml 100°C water for 30 min. Aqueous teas (and red wine) were filtered with 0.22-μm filters and firstly diluted 10 folds with external solution (normal drinking concentration, 1 g tea, 200 ml water) (Fig 1).

### Drugs

Tannic acid, quercetin, and terfinadine were purchased from Sigma-Aldrich Corp (St. Louis, MO, USA). The stock solutions were made in DMSO and were stored at -20°C. All solutions were freshly prepared from stock solutions before each experiment and kept from light exposure. The final concentration of DMSO was less than 0.1%.

### Statistical analysis

Currents were analyzed and fitted using Clampfit 10.2 (Molecular Devices, Sunnyvale, CA, USA) and Origin 7.5 (OriginLab Corp., Northampton, MA, USA) software. The concentration-response curve was fitted with logistic equation: *y* = *A*_*2*_+(*A*_*1*_–*A*_*2*_)/[1+(*x*/*x*_*0*_)^*p*^], where *y* is the response; *A*_*1*_ and *A*_*2*_ are the maximum and minimum response, respectively, *x* is the drug concentration, and *p* is the Hill coefficient. The current activation curves were generated by plotting the normalized tail current amplitudes against the step potentials and were fitted with a Boltzmann function: *y = A/{1 + exp[(V*_*h*_ *−V*_*m*_ *)/k]}*, where A is the maximal current amplitude, V_m_ is the test potential and k is the slope factor. All data are given as mean ± SEM. Differences between groups were assessed by Student’s *t* test or 1-way ANOVA. The differences were considered significant at *P* ≤ 0.05.

## Results

### Tannic acid inhibits the hERG currents and induces a rightward shift of the activation curve

**Fig 2:**
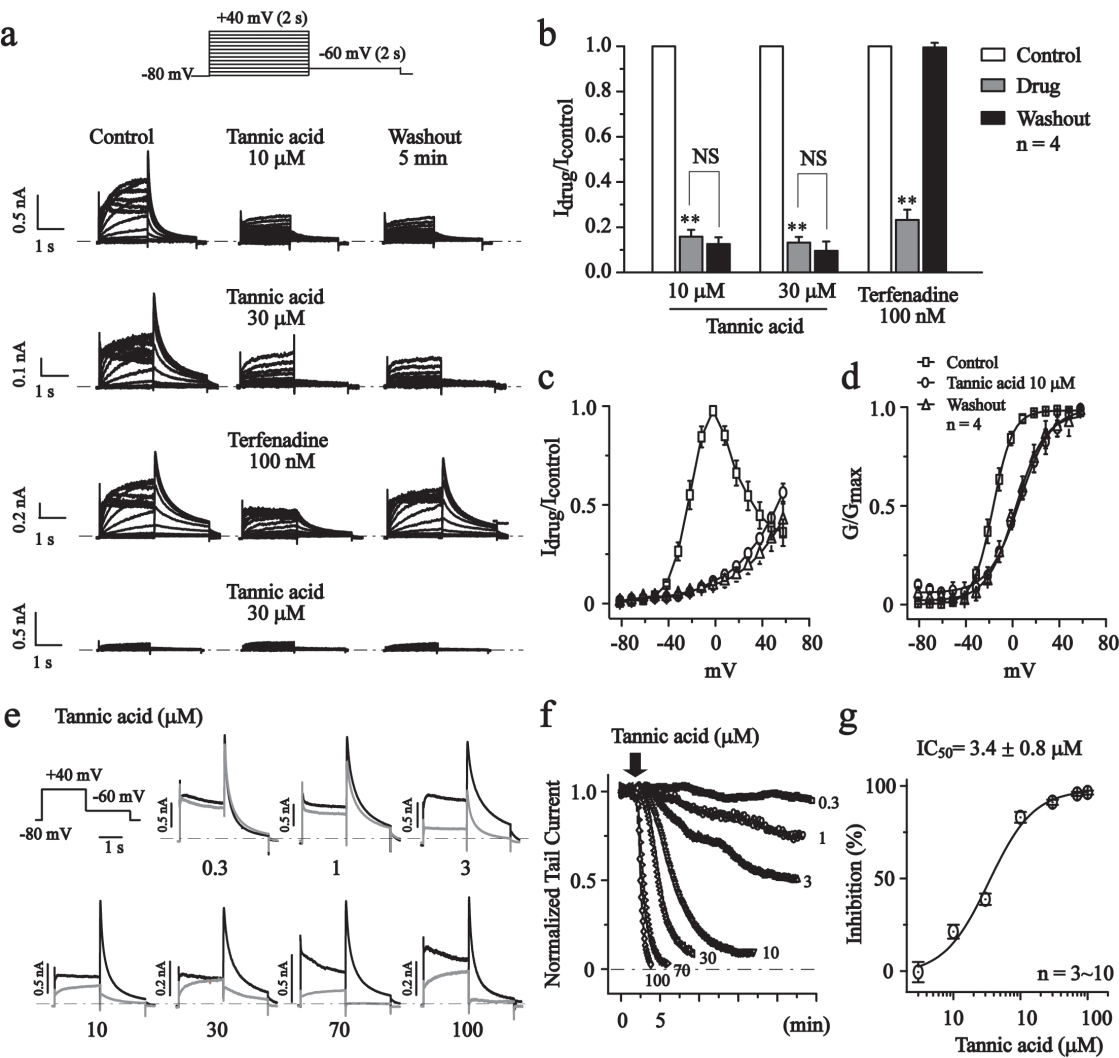
The effects of tannic acid on hERG currents. (a) Representative traces of hERG currents recorded using the voltage protocol indicated in the top panel. Dotted lines indicated the zero current level. The effects of tannic acid (10, 30 μM) or terfenadine (100 nM) and 5 min washout are shown. (b) Normalized peak tail current amplitudes at -60 mV (active potential: +40 mV). **P<0.01, NS, no significant, n = 4. (c) Normalized I-V curves of steady-state hERG active currents. (d) Normalized G-V curves constructed from the peak tail currents at -60 mV were fitted with the Boltzmann function. The V_1/2_ values were -15.4 ± 0.2 mV for the control, 7.8 ± 1.1 mV for tannic acid (10 μM), and 4.2 ± 1.0 mV for 5 min washout, n = 4. (e) The effects of different concentration of tannic acid were shown (black trace: control, gray trace: tannic acid). (f) The time courses of concentration-dependent modulation of the hERG currents recorded at -60 mV. (g) Concentration-response relationship of tannic acid on hERG currents (-60 mV, n = 3~10). The curve was fitted with the logistic function. The IC_50_ is 3.4 ± 0.8 μM.

We had a detailed investigation into the effect of tannic acid on hERG currents expressed in HEK293 cells. The hERG currents were measured with perforated patch clamp technique. Typical hERG currents were recorded with a standard protocol (Fig 2A, top). Tannic acid (10, 30 μM) quickly inhibited the hERG currents and the effect was irreversible upon washout (Fig 2A and 2B). The hERG currents were also inhibited by terfinadine (100 nM) (Fig 2A and 2B), a well established hERG blocker. While the HEK239 wild-type cells shown no typical hERG current (Fig 2A, bottom). Tannic acid (10 μM) significantly shifted the activation curve of hERG currents to more positive potentials (Δ23.2 mV, Fig 2D).

The concentration-response relationship of tannic acid was then established. For this investigation, hERG currents activated at +40 mV and -60 mV were measured at different concentrations of tannic acid. The concentration-response curves are shown in Fig 2G, in which the data are fitted with a logistic function. Tannic acid inhibited hERG currents with an IC_50_ value of 3.4 ± 0.8 μM (Fig 2G).

### Green tea Lung Ching inhibits the hERG currents and induces a rightward shift of the activation curve

**Fig 3:**
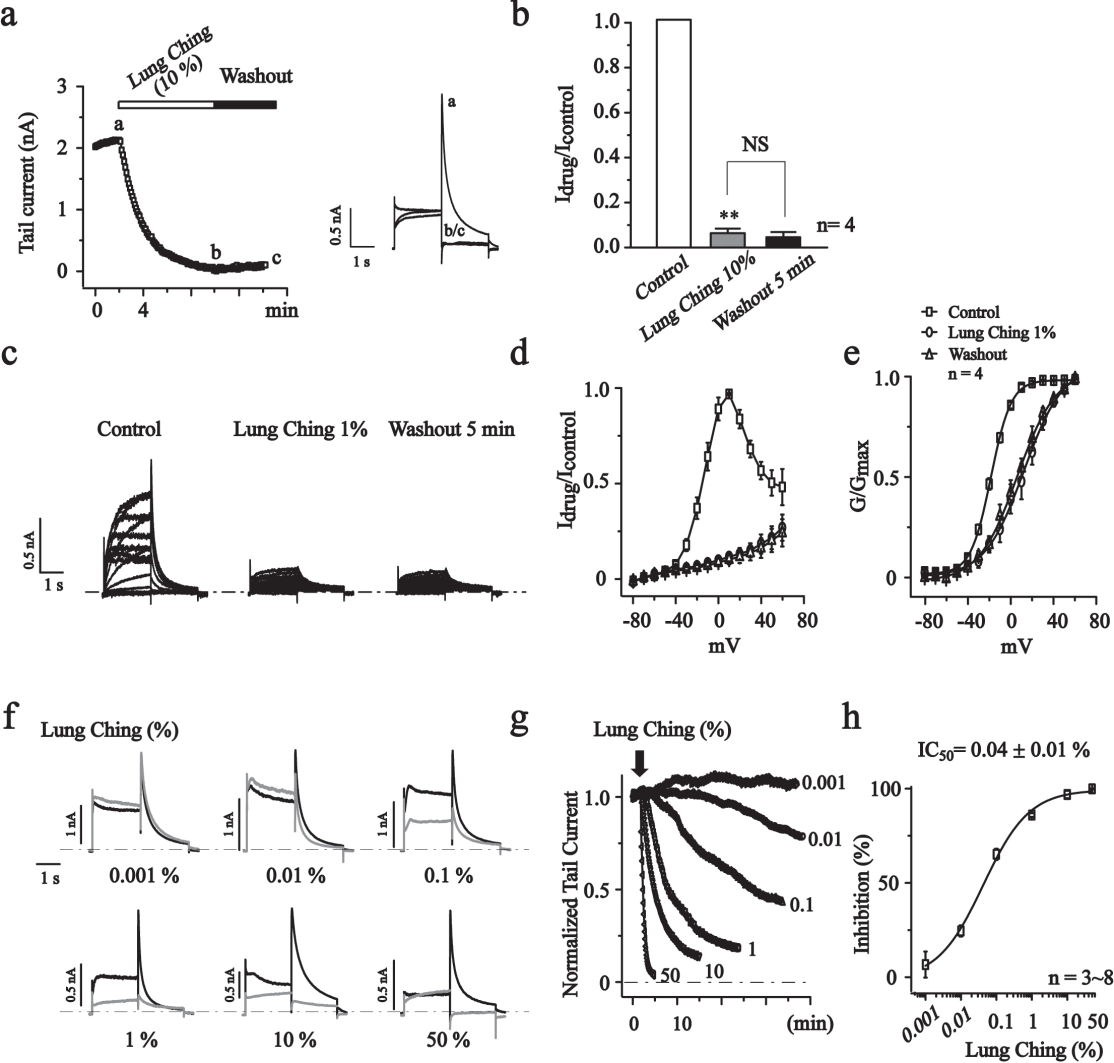
The effects of green tea Lung Ching on hERG currents. (a) The time courses for the effects of Lung Ching (10%) on hERG currents recorded at -60 mV. The right panel showed the current traces recorded at the times indicated by the letters. (b) Summary data of A. **P<0.01, NS, no significant. n = 4. (c) Representative traces of hERG currents recorded in the absence (Control) and in the presence of Lung Ching (1%) and after 5 min washout using a voltage step protocol indicated in Fig 2A. (d) Normalized I-V curves of steady-state hERG active currents. (e) Normalized G-V curves constructed from the peak tail currents at -60 mV were fitted with the Boltzmann function. The V_1/2_ values were -18.4 ± 0.2 mV for the control, 12.4 ± 1.1 mV for Lung Ching (1%), and 6.2 ± 0.8 mV for 5 min washout, n = 4. (f) The effects of different concentration of Lung Ching were shown (black trace: control, gray trace: Lung Ching). (g) The time courses of concentration-dependent modulation of the hERG currents recorded at -60 mV. (h) Concentration-response relationship of Lung Ching on hERG currents (-60 mV, n = 3~8). The curve was fitted with the logistic function. The IC_50_ is 0.04 ± 0.01%.

Tannic acid presents in teas with relatively high concentrations. Lung Ching (Dragon Well Tea), grown in the hillside of Hangzhou, is one of the most popular green tea in China. In this part, we invested the effect of Lung Ching on hERG currents expressed in HEK293 cells in details. At a concentration of 1%, Lung Ching potently inhibited the hERG currents and the effect was irreversible upon washout (Fig 3A and 3B). Lung Ching(1%) significantly shifted the activation curve to more positive potentials (Δ30.8 mV, Fig 3E). Lung Ching concentration-dependently inhibited hERG currents, with an IC_50_ of 0.04 ± 0.01% (Fig 3F–3H).

### Multiple types of teas inhibit the hERG currents

**Fig 4:**
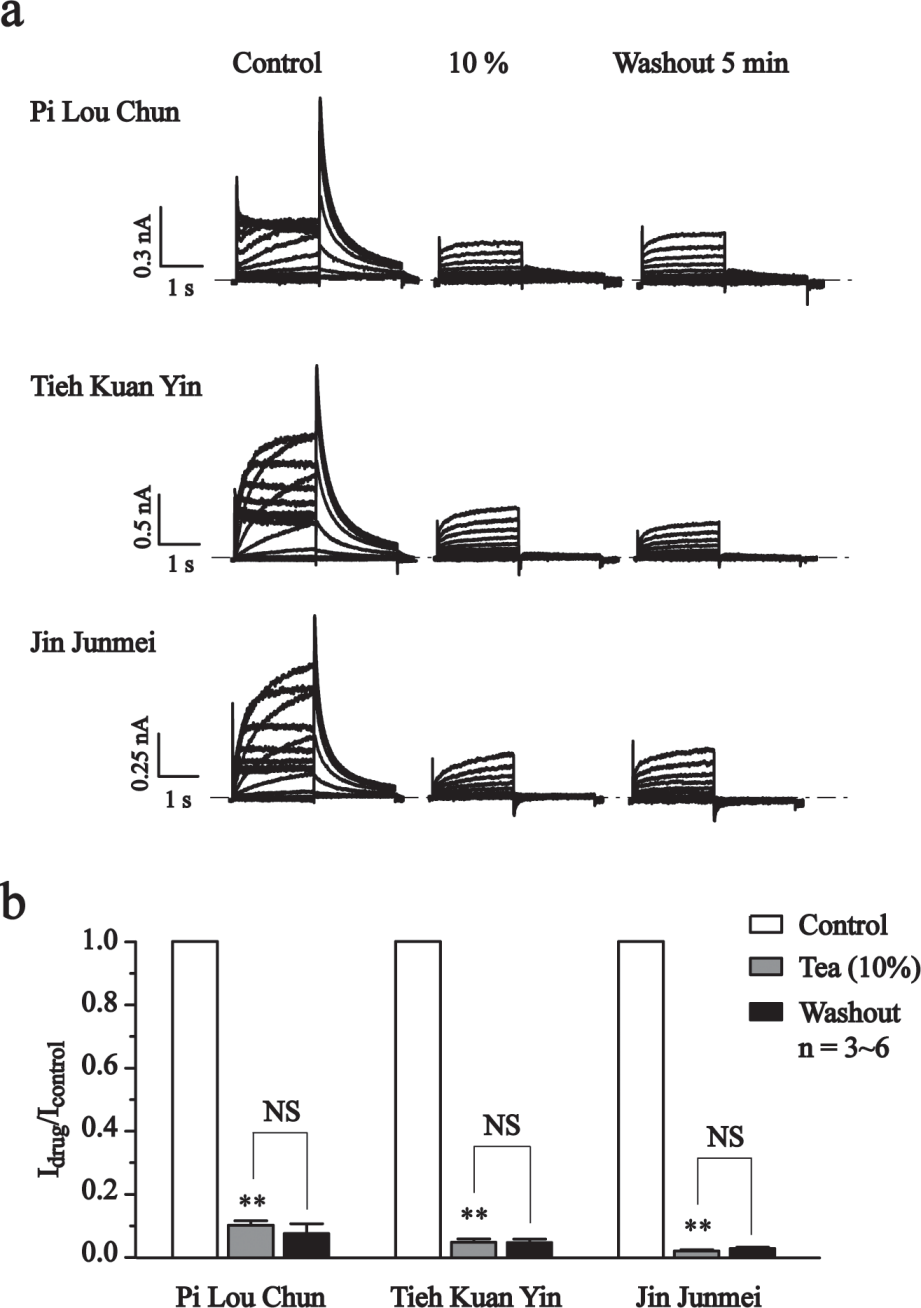
The effects of teas on hERG currents. (a) Representative traces of hERG currents recorded in the absence (Control) and in the presence of Pi Lou Chun, Tieh Kuan Yin or Jin Junmei (10%) and after 5 min washout using a voltage step protocol indicated in Fig 2A. (b) Normalized peak tail current amplitudes at -60 mV (active potential: +40 mV). **P<0.01, NS, no significant, n = 3~6.

While teas can be categorized into 3 types, depending on the level of fermentation, including green (non-fermented), oolong (semi-fermented) and black (fermented) tea. Thus, in this part, the effect of green tea Pi Lou Chun, oolong tea Tieh Kuan Yin and black tea Jin Junmei were tested on hERG currents expressed in HEK293 cells. As shown in Fig 4, Pi Lou Chun, Tieh Kuan Yin and Jin Junmei at concentration of 10% (1 g/2 L), potently inhibited the hERG currents and the effects were not reversible upon 5 min washout (Pi Lou Chun: 90 ± 1%, Tieh Kuan Yin: 95 ± 1%, Jin Junmei: 98 ± 1%).

### Tannic acid other than quercetin is responsible for the effects of teas on hERG currents

Flavonolds, riched in teas, were reported to cause a significant inhibition of hERG current [21]. To elucidate whether the inhibition effect of the teas on hERG currents might be because of their high content of flavonolds or tannic acid, the effects of flavonold quercetin and tannic acid were examined and compared with Lung Ching.

**Fig 5:**
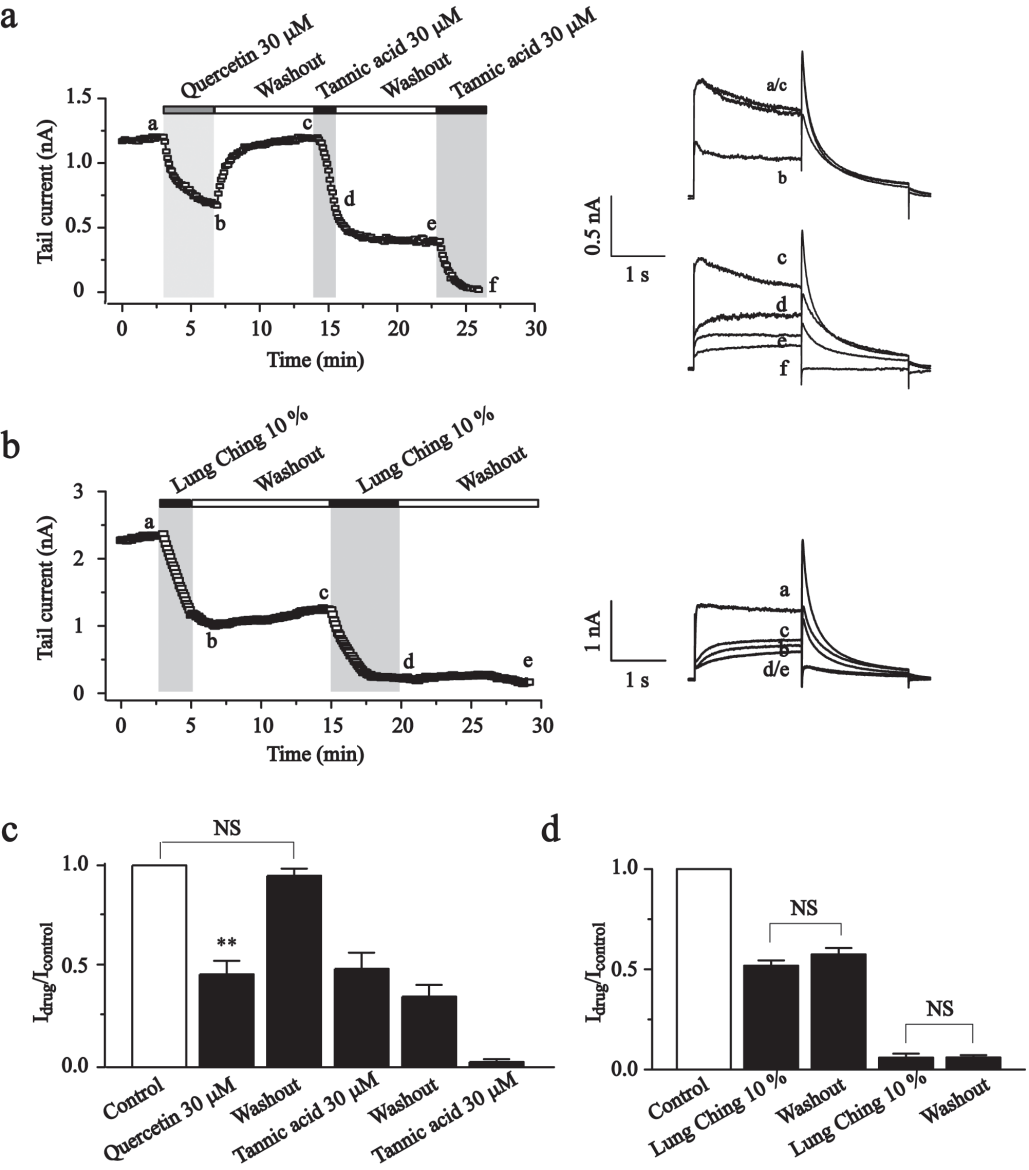
Tannic acid plays the key role in Lung Ching-induced hERG current inhibition. (a) The time courses for the effects of quercetin (30 μM), tannic acid (30 μM) and washout on the hERG currents recorded at -60 mV. The right panel showed the current traces recorded at the times indicated by the letters. (b) The time courses for the effects of Lung Ching (10%) and washout on the hERG currents recorded at -60 mV. The right panel showed the current traces recorded at the times indicated by the letters. (c) Summary for the effect of quercetin (30 μM), tannic acid (30 μM) and washout on the hERG currents. (d) Summary for the effect of Lung Ching (10%) and washout on the hERG currents. **P<0.01, NS, no significant.

At a concentration of 30 μM, quercetin exerted a mild inhibitory effect, reducing hERG currents by 54.1 ± 6.7%, and the effects were reversible (Fig 5A and 5C). In contrast, the effect of tannic acid (30 μM) and Lung Ching (10%, 1 g/2 L) were irreversible upon washout (Fig 5A–5D). These observations indicate that tannic acid other than quercetin may be responsible for the hERG currents inhibition effect of teas.

### Red wine inhibited the hERG currents and induces a rightward shift of the activation curve

**Fig 6:**
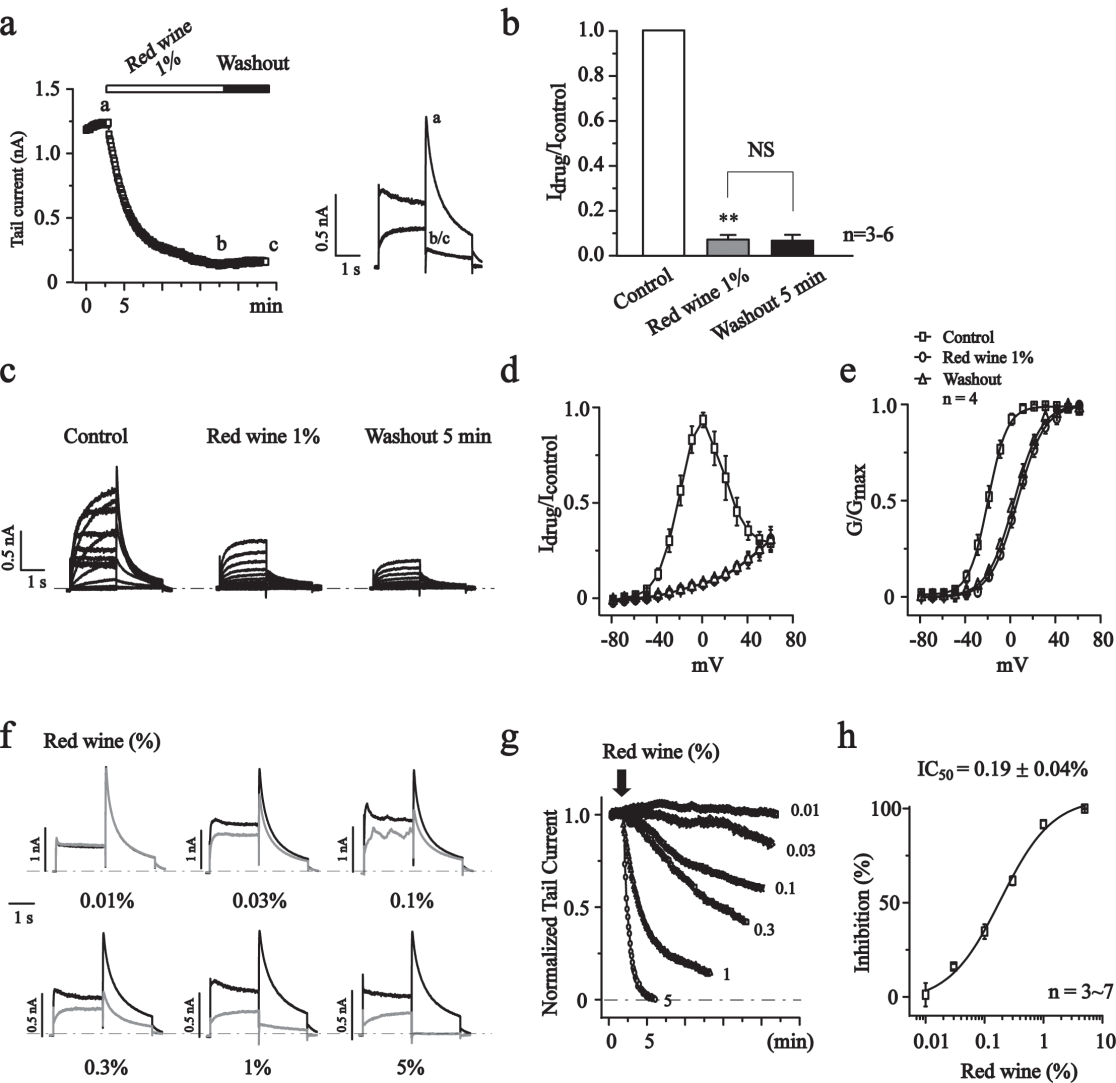
The effects of red wine on hERG currents. (a) The time courses for the effects of red wine (1%) on hERG currents recorded at -60 mV. The right panel showed the current traces recorded at the times indicated by the letters. (b) Summary data of A. **P<0.01, NS, no significant. n = 4. (c) Representative traces of hERG currents recorded in the absence (Control) and in the presence of red wine (1%) and after 5 min washout using a voltage step protocol indicated in Fig 2A. (d) Normalized I-V curves of steady-state hERG active currents. (e) Normalized G-V curves constructed from the peak tail currents at -60 mV were fitted with the Boltzmann function. The V_1/2_ values were -20.9 ± 0.3 mV for the control, 5.1 ± 0.4 mV for red wine (1%), and 2.4 ± 0.4 mV for 5 min washout, n = 4. (f) The effects of different concentration of red wine were shown (black trace: control, gray trace: red wine). (g) The time courses of concentration-dependent modulation of the hERG currents recorded at -60 mV. (h) Concentration-response relationship of red wine on hERG currents (-60 mV, n = 3~8). The curve was fitted with the logistic function. The IC_50_ is 0.19 ± 0.04%.

Tannic acid and related gallotannins are present in relatively high concentrations in red wines. We tested the effects of red wine for hERG inhibition. We found that red wine (Great Wall, China) significantly inhibited hERG currents at 1%, and the effect could not be washed out (Fig 6A and 6B). At a concentration of 1%, red wine potently inhibited the hERG currents at all test voltages (Fig 6C), and significantly shifted the activation curve to more positive potentials (Δ26.0 mV, Fig 6E).

The concentration-dependent effect of red wine on hERG currents was tested. As shown in Fig 6F and 6H, red wine concentration-dependently inhibited the hERG currents with an IC_50_ of 0.19 ± 0.04% and ~100% inhibition at higher concentrations. Red wine inhibits hERG channels with pharmacological characteristics similar to tannic acid and teas.

## Discussion

Tannic acid and related gallotannins are present in relatively high concentrations in teas and red wines. In the present study, we characterized the inhibitory effects of tannic acid, multiple teas and red wine on hERG currents. The results demonstrated that tannic acid may be one of the components responsible for this activity of teas and red wine. We demonstrated that tannic acid inhibited hERG currents with an IC_50_ of 3.4 μM and ~100% inhibition at higher concentrations. The inhibition of tannic acid on hERG currents are novel findings.

The hERG channels conduct the rapid delayed rectifier K^+^ current (I_*Kr*_) and mediate action potential repolarization in the heart, which could be recorded in myocardial cells freshly isolated from guinea pigs. But myocardial cell patch clamp recording are too pricy and difficult, which limited its widespread use in drug discovery and some other research. The hERG stably transfected HEK293 cells have been widely used for hERG channel modulation research for many years. The results in HEK293 cells have high coherence comparing with that obtained in myocardial cells from guinea pigs. So, in this research, we carried out our experiments in hERG stably transfected HEK293 cells.

Surprisingly, at a very low concentration of 100-fold dilution, green tea Lung Ching or red wine Great Wall inhibited hERG currents by ~90%, and significantly shifted the voltage dependent activation to more positive potentials (Figs 3 and 6), which showed very similar pharmacological characteristics with tannic acid. On the other hand, the effects of tested teas (Lung Ching, Pi Lou Chun, Tieh Kuan Yin and Jin Junmei) and red wine Great Wall on hERG currents were irreversible upon washout, which was similar to tannic acid’s effects, too (Fig 3B, Fig 4B and Fig 6B). Flavonoids have been shown to be responsible for some of the beneficial efficacy of green teas and red wines [22,23,24]. In our experiment, the effects of typical flavonoid quercetin were tested. Distinguish from teas and red wine, quercetin showed a reversible inhibition effect on hERG currents (Fig 5C) and significantly leftward shifted the voltage-activation curve (Zhang *et al*., unpublished observations). Thus effects of tannic acid on hERG currents seem to be identical to the effects of teas and red wine that are known to have an especially high content of tannins. On the other side, besides quercetin, our lab also found that multiple flavonolds, such as fisetin, luteolin and chrysin, caused a significant inhibition of hERG currents (Zhang *et al*., unpublished observations). Also it has been reported that morin, hesperetin, naringenin caused a significant inhibition of hERG currents [21], which was conformed by our data (S1 Fig). These data suggested that some of the flavonolds and maybe some other components synergized the effect of tannic acid in the tea or red wine.

Epidemiological studies have shown a negative association between tea consumptions and the incidence of cancers [25]. Studies demonstrated that tannic acid and related compounds suppresses the glucocorticoid-induced gene expression of mouse mammary tumor virus (MMTV) integrated into 34I cells [17,26]. In addition, tannic acid inhibits the proliferation of various cancer cell lines and induced cancer cell apoptosis and raises survival rate of mice bearing syngeneic tumors [19,20,27]. Recent evidence highlights the role of hERG channels in tumor cell biology: control of cell proliferation, invasiveness and neoangiogensis support the view that hERG may be used as a novel diagnostic and prognostic marker in human cancer as well as a target for anti-neoplastic therapies [4]. The hERG RNA and protein was found to be expressed in 67% and 82%, respectively, of cancerous cells, whereas this percentage was only 18% in non-cancerous tissues. And Specific hERG channel blockers can reduce cell proliferation [28]. Our data showed that tannic acid, teas and red wine were potent hREG channel blockers, which suggested that the anticancer activities of teas and red wine maybe attributed partially to their hERG inhibit properties.

## Conclusions

In summary, our data suggest that tannic acid is a potent hERG channel blocker, and this activity may contribute to its multiple beneficial effects (eg. antitumor properties). They also revealed a novel potential pharmacological basis of tea- and red wine-induced biology activities.

## Supporting Information

S1 Fig The effects of naringenin on hERG currents. (a) Chemical structure of naringenin. (b) The time courses for the effect of naringenin (100 μM) on hERG currents recorded at -60 mV.
(TIF)

## Abbreviations

hERG: human ether-à-go-go-related gene
HEK: human embryonic kidney
