# Supplementary figures and images for: Effects of Tannic Acid, Green Tea and Red Wine on hERG Channels Expressed in HEK293 Cells
Source: PLoS One. 2015 Dec 1;10(12):e0143797. doi: 10.1371/journal.pone.0143797 (PMC4666621; doi:10.1371/journal.pone.0143797)

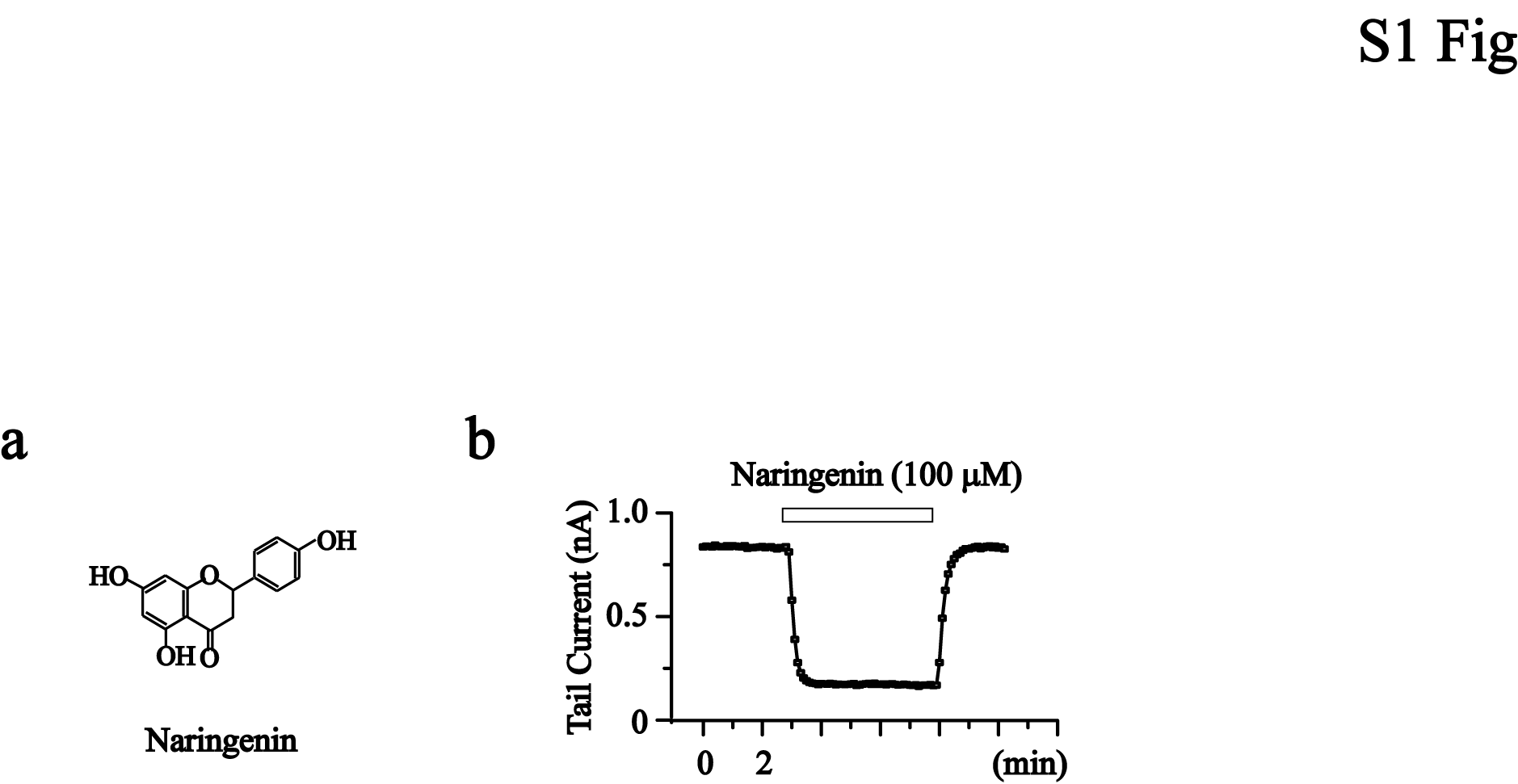

Supplement: S1 Fig — (a) Chemical structure of naringenin. (b) The time courses for the effect of naringenin (100 μM) on hERG currents recorded at -60 mV. (TIF) [file pone.0143797.s001.tif]
